# Quantitative hepatitis B core antibody level is associated with inflammatory activity in treatment-naïve chronic hepatitis B patients

**DOI:** 10.1097/MD.0000000000004422

**Published:** 2016-08-26

**Authors:** Min-Ran Li, Jian-Hua Lu, Li-Hong Ye, Xing-Li Sun, Yan-Hua Zheng, Zhi-Quan Liu, Hai-Cong Zhang, Yun-Yan Liu, Ying Lv, Yan Huang, Er-Hei Dai

**Affiliations:** Division of Liver Disease, The Fifth Hospital of Shijiazhuang, Hebei Medical University, Shijiazhuang, China.

**Keywords:** ALT, chronic hepatitis B, liver inflammation, quantitative anti-HBc

## Abstract

Previous studies have shown that hepatitis B core antibody (anti-HBc) levels vary during different phases of disease in treatment-naïve chronic hepatitis B (CHB) patients and can be used as a predictor of both interferon-α and nucleoside analogue therapy response. However, there is no information on the association between the quantitative serum anti-HBc (qAnti-HBc) level and liver inflammation in CHB patients. Therefore, we investigated these relationships in a large cohort of treatment-naïve CHB patients. A total of 624 treatment-naïve CHB patients were included in the study. The serum qAnti-HBc level was moderately correlated with ALT and AST levels (*P* < 0.001) in both hepatitis B e antigen-positive (HBeAg [+]) and HBeAg-negative (HBeAg [−]) CHB patients. CHB patients with no to mild inflammation (G0–1) had significantly lower serum qAnti-HBc levels than patients with moderate to severe inflammation (G2–4) (*P* < 0.001). Receiver operating characteristic analysis suggested that a serum qAnti-HBc cut-off value of 4.36 log_10_ IU/mL provided a sensitivity of 71.68%, specificity of 73.81%, positive predictive value of 78.43%, and negative predictive value of 66.24% in HBeAg (+) CHB patients with moderate to severe inflammation (G≥2). A cut-off value of 4.62 log_10_ IU/mL provided a sensitivity of 54.29%, specificity of 90.00%, positive predictive value of 95.00%, and negative predictive value of 36.00% in HBeAg (−) CHB patients with moderate to severe inflammation (G≥2). Serum qAnti-HBc levels were positively associated with liver inflammation grade. Furthermore, we identified optimal serum qAnti-HBc cut-off values for the prediction of inflammation activity in both HBeAg (+) and HBeAg (−) treatment-naïve CHB patients.

## 1 Introduction

Hepatitis B virus (HBV) is one of the major human pathogens that cause severe liver disease, including liver cirrhosis and hepatocellular carcinoma. Approximately 2 billion people are infected with HBV worldwide, 350 million of whom are chronic HBV carriers, and HBV infection causes over 600,000 deaths each year.^[1,2]^ In 1965, HBV infection was first discovered when Blumberg identified hepatitis B surface antigen (HBsAg).^[3]^ In 1979, HBV DNA was first sequenced by Galibert, which initiated HBV genomic research.^[4]^ Researchers have been making great progress in elucidating the constitution of the HBV genome, the life cycle of HBV, and the structural and biological traits of HBV antigens.

HBV consists of an external envelope (HBsAg) and an inner core (hepatitis B core antigen, HBcAg). Hepatitis B core antibody (anti-HBc) is generally formed during an infection with HBV, which often persists throughout the lifetime. As one of the most classical serological markers of HBV infection,^[5]^ anti-HBc has been widely used in clinical diagnosis or blood screening combined with HBsAg.^[1]^ One positive attribute of anti-HBc is that it is considered to be an indicator of both past and persistent HBV infection. Due to the limitation of quantitative detection technology and the lack of international standardization, the clinical significance of the anti-HBc quantitative (qAnti-HBc) level remains largely unknown.

Recently, a novel diagnostic immunoassay procedure for qAnti-HBc using homogeneous purified full-length HBcAg capsids obtained from Escherichia coli was developed to quantify serum anti-HBc levels.^[6]^ Based on the new quantitative method and standard information derived from World Health Organization reports,^[7]^ it was reported that qAnti-HBc levels vary during different phases of chronic hepatitis B (CHB) in treatment-naïve patients.^[8,9]^ Furthermore, it was demonstrated that the baseline levels of qAnti-HBc represented a new potential predictor of treatment response in both interferon-α and nucleoside analogue therapies.^[10,11]^ These findings highlight the clinical value of qAnti-HBc levels in CHB patients. However, the relationship between qAnti-HBc and liver inflammation activity in treatment-naive CHB patients remains unknown.

## 2 Materials and methods

### 2.1 Patients

From 2012 to 2015, consecutive CHB patients were assessed at the Fifth Hospital of Shijiazhuang, Hebei, China. The inclusion criteria were as follows: HBsAg-positive for at least 6 months, treatment-naïve, and scheduled for liver biopsy. The exclusion criteria were as follows: decompensated liver cirrhosis, hepatocellular carcinoma, liver transplantation, coinfections (hepatitis C virus, hepatitis D virus, and human immunodeficiency virus), causes of liver disease other than HBV, and immunosuppressive treatment. Liver biopsies and serum samples were obtained on the same day in all cases.

The study was in compliance with the Helsinki Declaration and was approved by the Medical Ethics Committee of The Fifth Hospital of Shijiazhuang. All the enrolled patients gave their written informed consent.

### 2.2 Laboratory measurements

Serum ALT levels were determined using a Hitachi 7600 (HITACHI, Japan) automated biochemistry analyzer. Serum HBV DNA levels were measured by real time fluorescence quantitative polymerase chain reaction assays on an ABI 7500 (Applied Biosystems), and the lowest limit of detection was 500 IU/mL. Serum HBsAg titers were quantified using an Elecsys HBsAg II quant assay (Roche Diagnostics, Branchburg, NJ), with a dynamic range from 0.05 to 130000 IU/mL. Hepatitis B e antigen (HBeAg) and anti-HBe were detected using Architect assays (Abbott Laboratories, North Chicago, Illinois). The serum qAnti-HBc level was measured using a newly developed double-sandwich immunoassay (Wantai, Beijing, China) that was calibrated using the World Health Organization standard (NIBSC, UK). The HBV genotype was assessed by sequencing. Liver inflammatory was assessed using the Scheuer scoring system, with the inflammatory grade measured on a scale of 0–4.^[12]^

### 2.3 Statistical analysis

Categorical variables were expressed as counts and percentages and were analyzed using the χ^2^ or Fisher's exact test. Continuous variables are presented as the means ± standard deviation (SD) or median (interquartile range). Student *t* test, 1-way analysis of variance or the Mann–Whitney *U* test was used for statistical comparisons where appropriate. Correlations of qAnti-HBc with ALT, HBV DNA, and other parameters were assessed using Spearman's method. Receiver operating characteristic (ROC) curves and areas under the ROC (AUROC) curves were calculated to evaluate the diagnostic accuracy of qAnti-HBc for liver inflammation activity. *P* values of less than 0.05 were considered statistically significant. Statistical analyses were performed using SPSS ver. 17.0 software (SPSS, Chicago, IL).

## 3 Results

### 3.1 Patient characteristics

A total of 624 treatment-naïve CHB patients were enrolled in the study, 489 of whom were HBeAg-positive (HBeAg [+]) and 135 of whom were HBeAg-negative (HBeAg [−]). The demographic, clinical, and histological characteristics of the patients are presented in Table 1. There were more males (68.59%) than females. The HBeAg (−) patients were older than the HBeAg (+) patients (*P* < 0.001). Compared with the HBeAg (−) patients, the HBeAg (+) patients had significantly higher platelet (PLT), HBV DNA, and HBsAg values but had significantly lower total bilirubin and qAnti-HBc levels. HBeAg (+) patients exhibited similar serum levels of alanine aminotransferase (ALT) and aspartate aminotransferase (AST) to HBeAg (−) patients. HBV genotype C was significantly more prevalent among all patients, and there were no significant differences in the frequency of the HBV genotype between HBeAg (+) and HBeAg (−) patients (*P* = 0.23). There were significant differences in the frequency of the different grades of liver inflammation between the two patient groups. The proportion of patients with severe portal/periportal inflammation and lobular inflammation in the HBeAg (−) group was greater than that in the HBeAg (+) group.

**Table 1:**
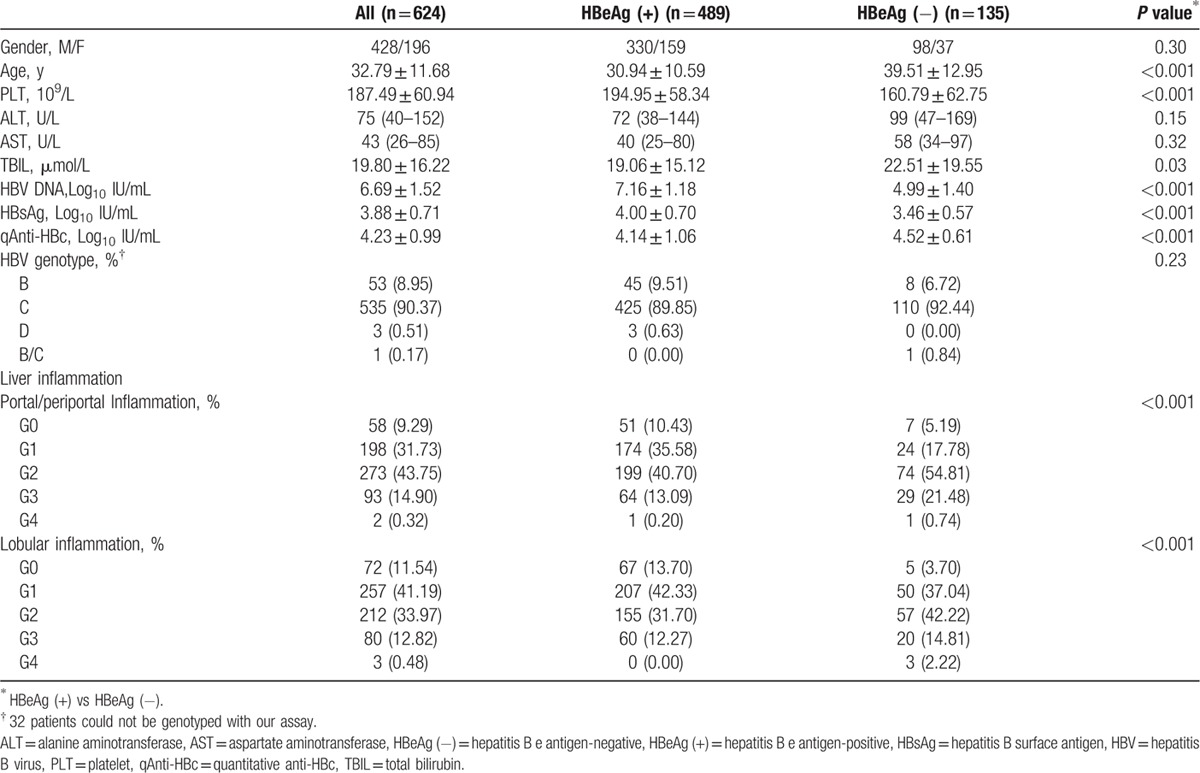
Patient characteristics.

### 3.2 Correlation of serum qAnti-HBc with ALT and other clinical parameters

The correlation of serum qAnti-HBc with ALT and other clinical parameters is shown in Table 2. Among the HBeAg (+) CHB subjects, the Spearman correlation analysis showed that the serum qAnti-HBc level was only moderately correlated with ALT (*R* = 0.559, *P* < 0.001) and AST (*R* = 0.580, *P* < 0.001). Additionally, there was a positive correlation observed in the HBeAg (−) CHB subjects between serum qAnti-HBc and ALT (*R* = 0.400, *P* < 0.001) and between serum qAnti-HBc and AST (*R* = 0.411, *P* < 0.001).

**Table 2:**
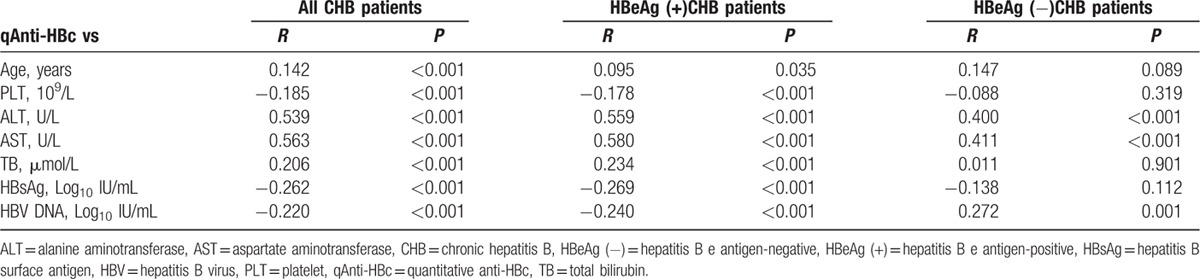
Correlation between qAnti-HBc levels with other clinical parameters in CHB subjects.

**Figure 1:**
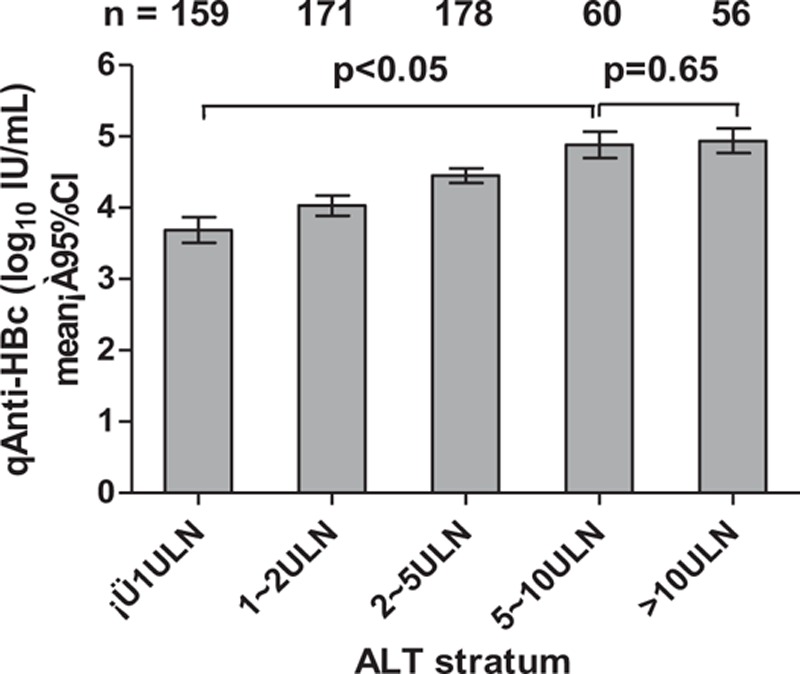
Mean serum qAnti-HBc levels in all CHB subjects according to the ALT stratum. ALT = alanine aminotransferase, CHB = chronic hepatitis B, qAnti-HBc = quantitative anti-HBc.

The correlation between serum qAnti-HBc and ALT was further analyzed among all the CHB subjects. Among the subjects in the first 5 ALT strata (5 × the upper limit of normal [ULN]), the mean qAnti-HBc level successively increased with increasing ALT level (*P* < 0.05). When the ALT level reached 5 times the ULN, it plateaued (*P* = 0.65) (Fig. 1).

### 3.3 Genotype analysis

Thirty-two patients could not be genotyped with our assay and were excluded from further analysis. Three other patients who were infected with genotype D and 1 other patient who was infected with the B/C mixed genotype were also excluded from the analysis. In the overall population with an identified genotype, only 8.95% of patients were infected with HBV genotype B. No significant difference was observed between the genotype B and C groups with regard to ALT, AST, HBV DNA, HBsAg, or qAnti-HBc levels (*P* > 0.05). The distributions of HBV genotypes were stratified according to liver inflammation grade. The frequencies of the different portal/periportal inflammation grades between the 2 groups were significantly different (*P* = 0.003), and the proportion of patients with severe portal/periportal inflammation in the genotype C group was greater than that in the genotype B group. However, the frequencies of the different lobular inflammation grades between the two groups were similar (*P* = 0.19) (Table 3).

**Table 3:**
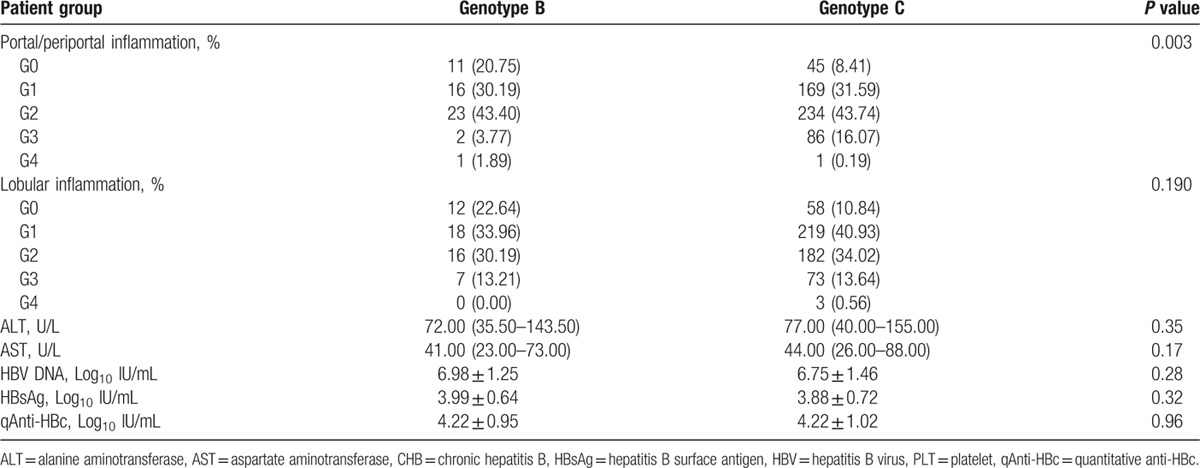
Inflammation severity and HBV serum markers according to HBV genotype in CHB patients.

### 3.4 Correlation between serum qAnti-HBc and inflammation grade

**Figure 2:**
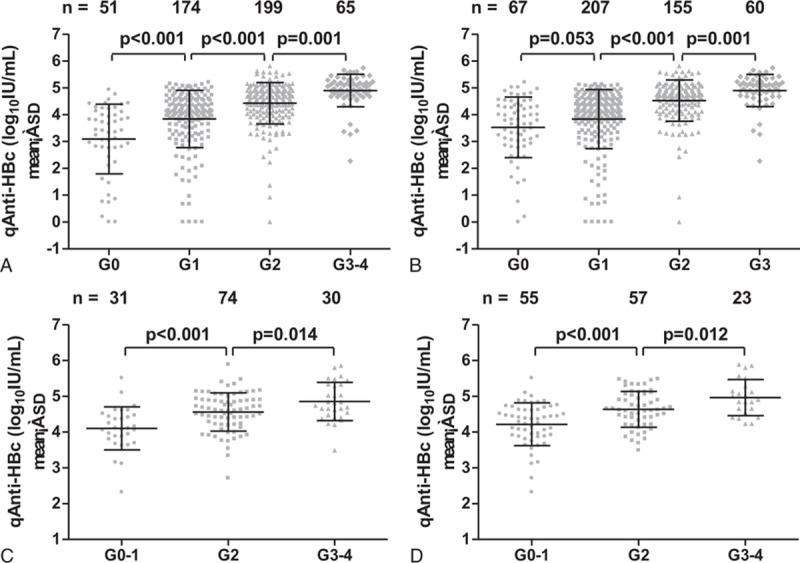
Correlation between serum qAnti-HBc levels and severity of portal/periportal and lobular inflammation in HBeAg (+) (A/B) and HBeAg (−) CHB patients (C/D). CHB = chronic hepatitis B, HBeAg (−) = hepatitis B e antigen-negative, HBeAg (+) = hepatitis B e antigen-positive, qAnti-HBc = quantitative anti-HBc.

Serum qAnti-HBc levels in HBeAg (+) and HBeAg (−) patients were stratified according to the level of liver inflammation. Among the HBeAg (+) CHB patients, the mean levels of qAnti-HBc for different grades of portal/periportal inflammation were as follows: G0 (3.10 ± 1.30 log_10_IU/mL), G1 (3.84 ± 1.07 log_10_IU/mL), G2 (4.43 ± 0.77 log_10_IU/mL), and G3–4 (4.90 ± 0.61 log_10_IU/mL); the mean levels of qAnti-HBc for different grades of lobular inflammation were as follows: G0 (3.53 ± 1.13 log_10_IU/mL), G1 (3.84 ± 1.10 log_10_IU/mL), G2 (4.53 ± 0.77 log_10_IU/mL), and G3 (4.90 ± 0.60 log_10_IU/mL). The mean qAnti-HBc level in G0/G1 subjects was significantly lower (*P* < 0.001) than that in G2 and G3/G3–4 subjects, and there was also a significant difference between G2 and G3/G3–4 subjects (*P* = 0.001) (Fig. 2A and 2B). Among the HBeAg (−) patients, the mean levels of qAnti-HBc for different grades of portal/periportal inflammation were as follows: G0–1 (4.10 ± 0.60 log_10_IU/mL), G2 (4.56 ± 0.54 log_10_IU/mL) and G3–4 (4.86 ± 0.54 log_10_IU/mL); the mean levels of qAnti-HBc for different grades of lobular inflammation were as follows: G0–1 (4.22 ± 0.60 log_10_IU/mL), G2 (4.64 ± 0.50 log_10_IU/mL), and G3–4 (4.97 ± 0.51 log_10_IU/mL). The trend in qAnti-HBc levels among different grades of liver inflammation was similar between the HBeAg (−) and HBeAg (+) CHB patients (Fig. 2C and 2D).

### 3.5 Use of the qAnti-HBc level to distinguish between liver inflammation grades

Based on the grade of portal/periportal and lobular inflammation, data from the HBeAg (+) and HBeAg (−) CHB patient groups were stratified into two groups: no to mild inflammation (G0–1) and moderate to severe inflammation (G2–G4) groups. HBV markers and ALT were calculated for the 2 groups (Table 4). HBeAg (+) patients with no to mild inflammation had significantly lower serum qAnti-HBc and ALT levels compared with patients with moderate to severe inflammation (*P* < 0.001). However, HBV DNA was significantly higher in HBeAg (+) patients with no to mild inflammation (*P* < 0.001). Among the HBeAg (−) patients, the no to mild inflammation group had significantly lower serum qAnti-HBc and ALT as well as HBV DNA, compared with the patients in the moderate to severe inflammation group (*P* < 0.001).

**Table 4:**
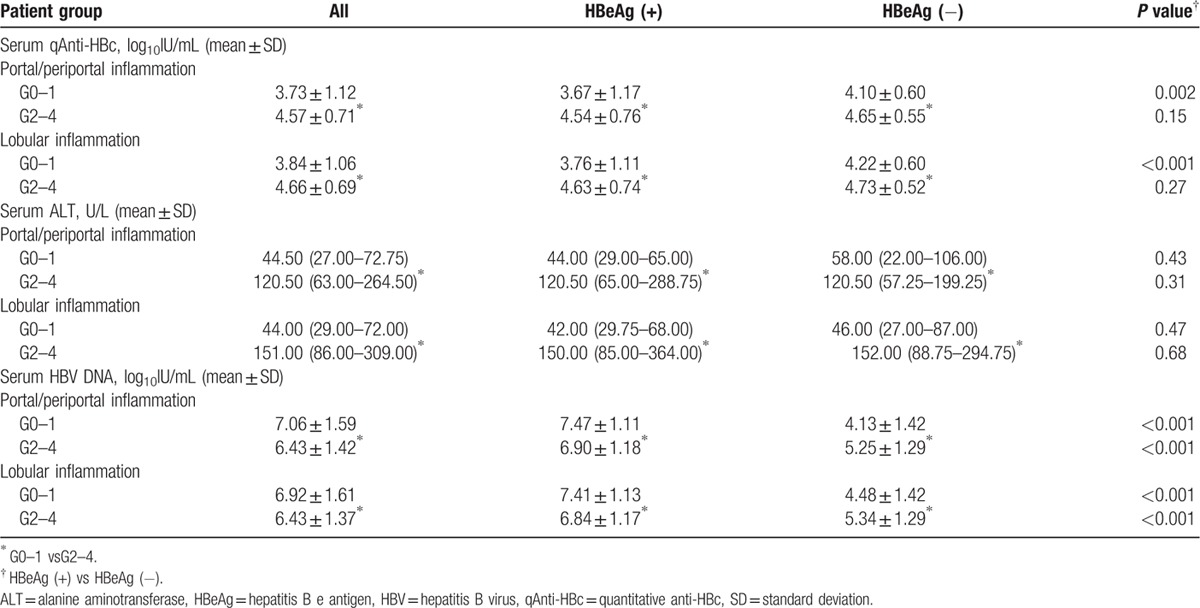
HBV serum markers and ALT levels according to HBeAg status and inflammation grade.

Compared with the corresponding ranges in HBeAg (−) patients, HBeAg (+) patients with G0–1 liver inflammation exhibited a significantly lower serum qAnti-HBc level (*P* < 0.01). In contrast, the HBV DNA levels were significantly higher in HBeAg (+) patients compared with HBeAg (−) patients, regardless of inflammation grade (*P* < 0.001). There was no significant difference in serum qAnti-HBc in the G2–G4 group or ALT levels in both the G0–1 and G2–G4 groups between HBeAg (+) and HBeAg (−) patients (*P* > 0.05).

### 3.6 ROC analysis

**Figure 3:**
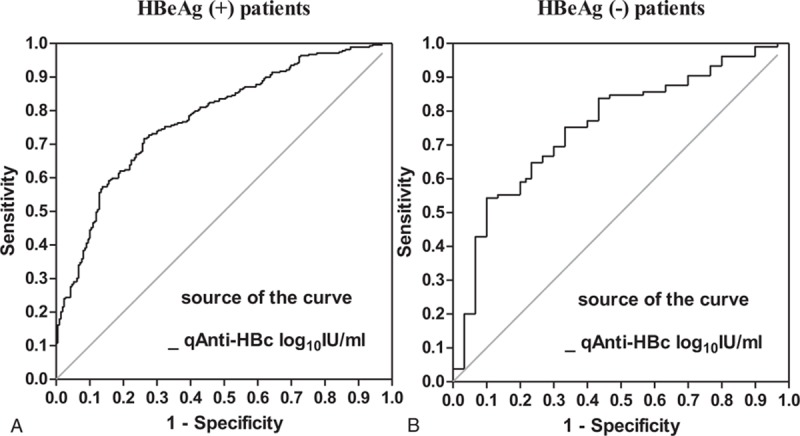
Receiver-operating characteristic curve of qAnti-HBc levels used to distinguish moderate to severe inflammation in HBeAg (+) (A) and HBeAg (−) (B) CHB patients. CHB = chronic hepatitis B, HBeAg (−) = hepatitis B e antigen-negative, HBeAg (+) = hepatitis B e antigen-positive, qAnti-HBc = quantitative anti-HBc.

ROC analysis was performed to distinguish moderate to severe inflammation by qAnti-HBc level among HBeAg (+) and HBeAg (−) CHB patients. In HBeAg (+) CHB patients, the area under the receiver-operating characteristic (areas under the ROC) curve of qAnti-HBc was 0.779 (95%CI: 0.738–0.820) for moderate to severe inflammation. The cut-off value of 4.36 log_10_IU/mL for moderate to severe inflammation had a sensitivity of 71.68%, specificity of 73.81%, positive predictive value of 78.43%, and negative predictive value of 66.24%. In HBeAg (−) CHB patients, the AUROC curve was 0.755 (95%CI: 0.659–0.851). The cut-off value was 4.62 log_10_ IU/mL, with a sensitivity of 54.29%, specificity of 90.00%, positive predictive value of 95.00%, and negative predictive value of 36.00% (Fig. 3).

## 4 Discussion

Qualitative detection of the five serological markers of HBV is routinely used as diagnostic and/or prognostic indicators of acute or chronic HBV infection. The presence of HBsAg is the most common marker of HBV infection, and HBeAg is used as an ancillary marker, primarily to indicate active HBV replication associated with progressive liver disease, whereas the presence of anti-HBc is considered to be an indicator of both past and persistent HBV infection. Along with the development of quantitative technology, researchers investigated the new clinical significance of HBV serological markers. The quantification of serum HBsAg titers could add value to HBV DNA quantification and could improve treatment monitoring.^[13]^ Previous studies revealed that serum qAnti-HBc levels are closely related to the host immune status and are strongly associated with hepatitis activity in CHB patients. Song LW et al^[9]^ showed that the mean qAnti-HBc levels in patients in the immune clearance phase and HBeAg-negative hepatitis phase were significantly higher than those in patients in both the immune tolerance phase and the low-replicative phase. They also demonstrated that serum qAnti-HBc levels were positively correlated with ALT levels. Another study showed similar results.^[8]^ However, there is a lack of direct evidence from liver biopsies to confirm these results. In the present study, we investigated the relationship between serum qAnti-HBc levels and liver inflammation grades.

To our knowledge, this is the first study to investigate the relationship between serum qAnti-HBc levels and liver inflammation activity in treatment-naïve CHB patients. All of the included patients were treatment-naïve. The results showed that serum qAnti-HBc levels, ALT, and liver inflammation activity were closely related to each other. First, we found that serum qAnti-HBc levels were moderately correlated with ALT and AST levels in both HBeAg (+) and HBeAg (−) CHB patients. Furthermore, we found a significantly lower level of qAnti-HBc in patients with no to mild inflammation (G0–1) than in those with moderate to severe inflammation (G2–4). We also observed increased serum levels of ALT to be associated with increased inflammation severity. While the exact underlying mechanism of the positive correlation between serum qAnti-HBc level, serum ALT level, and inflammation severity remains to be examined, a possible mechanistic explanation for this association is that all of these features are determined by the host's immune responses. HBV is not directly cytopathic to hepatocytes, and hepatocellular damage observed during chronic HBV infections appears to be primarily caused by the host's immune responses to the virus. Tissue-damaging inflammation occurs when the host's immune system attacks liver cells. ALT and HBcAg can be released from damaged infected hepatocytes into the bloodstream. An increase in the serum ALT level causes potent antigenic stimulation of B cells, resulting in an increase in the serum qAnti-HBc level.

However, serum qAnti-HBc titers reached a plateau and no longer showed a correlation with ALT levels when ALT levels were greater than 5 × ULN. These results are consistent with those of a previous study^[9]^ and suggest that the immune system may not be overactivated without limitation when ALT is higher than 5 × ULN.

Treatment guidelines for CHB by international liver associations (American Association for the Study of Liver Diseases and Asian Pacific Association for the Study of the Liver) recommend that CHB patients begin antiviral treatment when ALT is consistently more than 2 × ULN. When serum ALT levels range between normal and 2 × ULN, liver inflammation activity should be moderate to severe.^[14,15]^ However, liver biopsy is an invasive procedure. Therefore, we identified the grade of liver inflammation according to qAnti-HBc level. Our analyses established a qAnti-HBc level of 4.36 log_10_ IU/mL or more as an optimal cut-off value for identifying HBeAg (+) subjects with moderate to severe inflammation and 4.62 log_10_ IU/mL or more for HBeAg (−) subjects. The above results revealed a potential role of qAnti-HBc levels in reflecting different levels of liver inflammation in chronic HBV infection. The baseline qAnti-HBc levels would be convenient for application in clinical practice, especially for subjects with ALT 2 × ULN or less before initiating antiviral treatment to optimize the antiviral treatment. However, qAnti-HBc alone is not sufficient to accurately predict liver inflammation grade, and it should be combined with other parameters. These issues will be addressed in our future studies.

Our study also had a few limitations. Only 4 patients with G4 inflammation were included, and they were merged into the G3 group for the data analysis. In cases of severe inflammation in clinical practice, most physicians would first choose general liver protection therapy and then consider liver biopsy. The level of serum qAnti-HBc in CHB patients with G4 inflammation requires further study. Additionally, our study only included patients with genotypes B and C, while data for patients with genotypes A and D, which are prevalent in Europe, need to be further investigated.

In conclusion, our study demonstrated that serum qAnti-HBc levels were significantly correlated with liver inflammation grade in treatment-naïve CHB patients. Furthermore, we defined a serum qAnti-HBc cut-off for the identification of moderate to severe inflammation in HBeAg (+) and HBeAg (−) CHB patients.
